# “Alcohol intoxication by proxy on a NICU” - a case report

**DOI:** 10.1186/s12887-022-03567-w

**Published:** 2022-09-02

**Authors:** Ulrike Wurst, Benjamin Ackermann, Wieland Kiess, Ulrich Thome, Corinna Gebauer

**Affiliations:** 1 Department for Neonatology, Hospital for Children and Adolescents, University Hospital Leipzig, Liebigstrasse 20a, 04103 Leipzig, Germany; 2 Hospital for Children and Adolescents, Department of Women and Child Health, University Hospital Leipzig, Liebigstrasse 20a, 04103 Leipzig, Germany

**Keywords:** Premature infant, Ethanol intoxication, mother’s milk, Communication, Child safeguarding

## Abstract

**Background:**

Ethanol intoxications in newborns are generally due to false preparation of formula with alcoholics or alcohol consumption by the breastfeeding mothers. Rarely, intoxications occur in hospitalized newborns, e.g., from excessive use of alcoholic hand sanitizers. We herein report a strange case of acute ethanol intoxications in our NICU.

**Case presentation:**

An extremely premature infant (23 0/7 weeks gestational age, birthweight 580 g) suffered from repeated life-threatening events with hemodynamic compromise, apnea, and lactic acidosis while being treated in our neonatal intensive care unit (NICU). Symptomatic treatment with intravenous fluids and, if necessary, intubation and catecholamine therapy led to recovery after several hours each time. The episodes eventually turned out to be severe ethanol intoxications brought about by breast milk contaminated with ethanol. The breast milk was supplied by the infant’s mother, who consumed non-trivial amounts of alcohol to build up her strength and make herself produce more milk, which was recommended to her by a family member. Additionally, she supplemented her own mother’s milk with cow’s milk because she was worried her baby was underserved with her milk. The mother admitted to this in intensive conversations with our team and a professional translator.

**Conclusions:**

This unique case underlines how different cultural dynamics can attribute to life-threatening events in the care of premature infants. It is important for us to emphasize that intensive communication and building a confident relationship with the parents of patients is essential to the work on NICUs. Child safeguarding issues and possibilities of intoxications have to stay in mind even in a supposedly safe space like the NICU.

## Background

Ethanol intoxications in newborns are rarely reported. Symptoms include tachycardia, flush, hypotonia, apathy, and apnea [1]. Intoxications are generally due to the faulty preparation of formula with alcoholic beverages or alcohol consumption by the breastfeeding mothers [2, 3]. Older studies report, that alcohol intake during breastfeeding was even considered to be beneficial and might promote lactation [2]. The prevalence of drinking alcoholic beverages postpartum ranges from 19 to 47% in different countries [4]. However, research has demonstrated an adverse effect of alcohol on the amount of produced milk by causing hormonal changes in the lactating mother [5]. Studies demonstrate that the maximum of alcohol concentration in the breastmilk is 30–60 minutes after maternal intake and is nearly as high as the concentration in maternal blood [2]. Rarely, intoxications occur in hospitalized newborns, e.g., from excessive use of alcoholic hand sanitizers or ethanol-containing medicinal preparations [6–8]. We herein report an unusual case of acute ethanol intoxication in our NICU.

## Case presentation

An extremely premature infant (23 0/7 weeks gestational age, birth weight 580 g) was born to consanguine parents (2nd degree cousins) of middle eastern origin and Yazidi ethnicity and admitted to the NICU of a German University Children’s Hospital. The family’s medical history was ordinary, with no history of any chronic medical conditions. Due to premature unstoppable labor, the baby was born vaginally without antenatal corticosteroids. The newborn suffered from severe respiratory distress syndrome and required substitution of surfactant four times and inhaled nitric oxide and high-frequency oscillatory ventilation. Gradual weaning and conversion to conventional ventilation finally allowed extubation on the 22nd day of life. Additionally, she showed bilateral subependymal hemorrhage (Papile classification grade 1) at admission, without progression. Nutrition was applied partly parenterally for 6 weeks and enterally via a nasogastric tube with pasteurized mother’s milk and donor human milk from the hospital’s milk bank, as well as hydrolyzed formula.

**Fig. 1:**
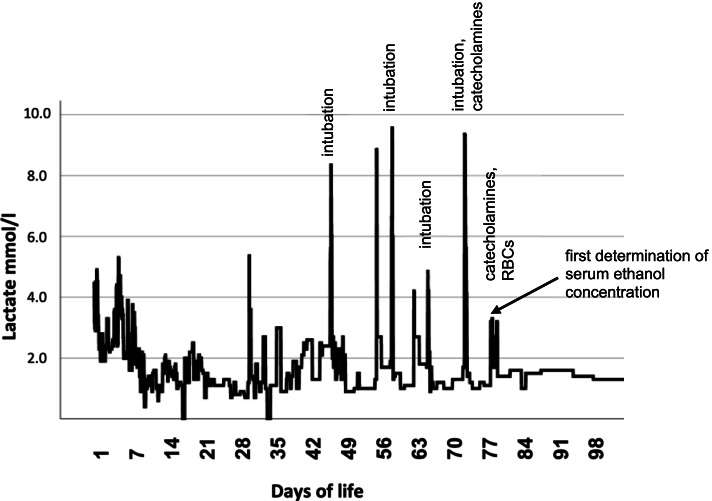
Measured lactate levels in the infant from 1st to 98th day of life. Visible peaks at different time points indicate lactate acidosis and suggesting episodes with ethanol intoxications. Notably, no further lactate acidosis occurred after finding out the cause of the condition after the 78th day of life

On the 45th day of life, paroxysmal apnea, hypotonia and coma occurred, preceded by tachycardia. Ultrasound revealed diminished renal and cerebral perfusion with zero flow or reverse flow. Echocardiography showed hypovolemia but no myocardial dysfunction. The infant was intubated, mechanically ventilated, and intravenous glucose-containing crystalloid fluids were applied. Gradual stabilization occurred within several hours; the infant was extubated and subsequently showed stable vital signs. A similar episode occurred on the 54th day of life, with the same dynamics and symptoms, again leading to intubation, ventilation, and need for high-volume fluid therapy. Additional diagnostic workup showed normal amplitude-integrated EEG with variations according to brain immaturity. Symptomatic treatment was extended by catecholamines and red blood cell transfusion as required. Noticeable was a specific pattern in the timely sequence of the symptoms, always beginning with tachycardia after a visit of the infant’s mother. Similar events recurred for another five times (days of life 57, 61, 64, 71, 77, and 78, Fig. 1).

### Laboratory findings

**Fig. 2:**
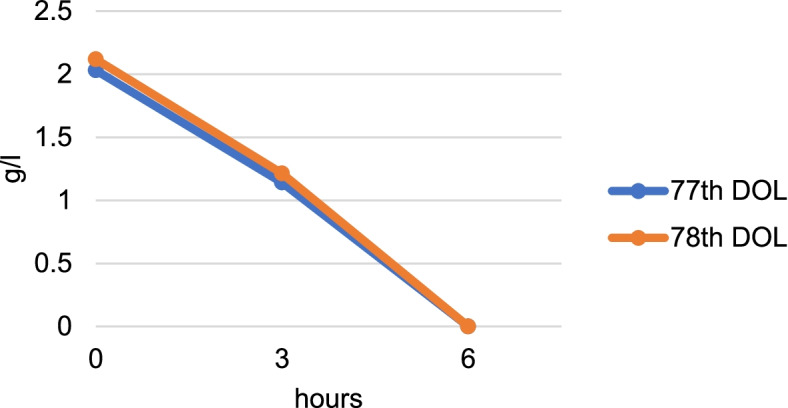
Decrease of serum ethanol concentrations measured in the infant during two episodes of lactic acidosis and clinical deprivation at initial symptomatic begin and after three and six hours. Notable are elevated initial serum levels and a rapid decrease after three hours to a non-detectable level after six hours under symptomatic treatment with intravenous fluids. *DOL: day of life*

Blood workup showed lactic acidosis (9.6 mmol/l at the maximum on day of life 57), but was inconclusive for signs of infection or bleeding. Metabolic workup showed no hyperammonemia, thyroid hormones (to exclude the differential diagnosis thyrotoxicosis) were normal. On the 77th day of life, diagnostic workup was extended by toxicologic analysis revealing a severe ethanol intoxication with highly elevated blood ethanol (2.0 g/l ≙1.62‰). The toxicologic screen in the urine was also highly positive for ethanol. Further analysis detected no additional drugs besides the prescribed medications. These findings were confirmed when another episode occurred on the next day with even higher serum ethanol concentrations of 2.1 g/l (≙1.7‰). We monitored serum ethanol concentrations in the blood with a swift decrease under symptomatic treatment (Fig. 2); we calculated the plasma half-live periods to 240 min and 236 min. Ethylglucoronid (ETG) was determined in the urine as a long-term marker, showing values above the cut-off of 25 mg/l, consistent with repeated ethanol intoxications.

**Fig. 3:**
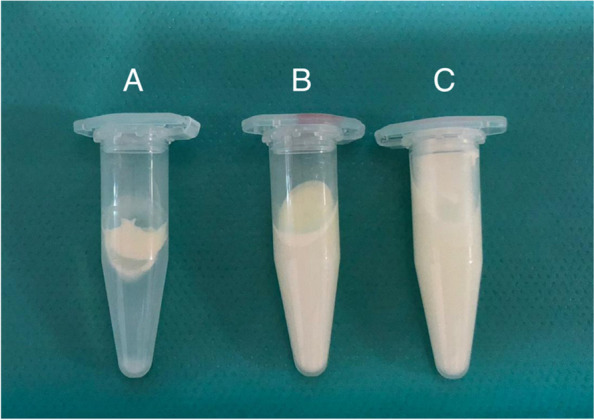
After noting a particular smell of the mother’s provided milk, centrifugation of three milk aliquots was performed. Obvious is the two-phase composition of donor human (**A**) milk in cream and whey, whereas both cow’s milk (**C**) and the infant’s mother’s milk (**B**) show no separation

**Fig. 4:**
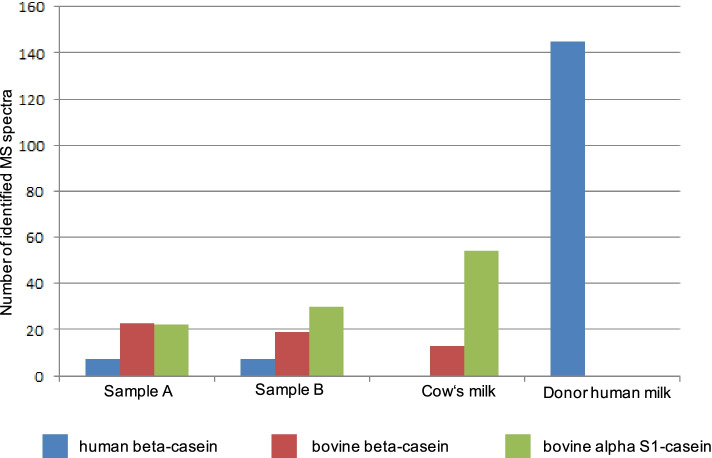
Mass spectral analysis of samples provided by the mother (samples **A**&**B**). Also demonstrated are typical spectra of cow‘s milk and human donor milk. As alpha-casein-S1 is a specific ingredient of cow’s milk and is not detectable in human milk (4th column), we proved the cocktail of cow‘s and human milk in our case

We excluded auto-brewery syndrome (fermentation of ingested carbohydrates to alcoholic products by microbes in the body, e.g. *Saccharomyces cerevisiae*) by repeated microbiological stool analysis as cause of the ethanol intoxication. Furthermore, iatrogenic ethanol intoxication by administration of medication in alcohol-based solutions was ruled out by carefully assessing the given medications. However, analysis of the mother’s milk to elucidate the source of the intoxications revealed an ethanol content of 2%. Furthermore, it was noticed that the smell of the milk provided by the mother resembled that of conventional cow’s milk. We centrifugated samples of the milk and found no separation into cream and whey in the milk provided by the mother. The milk therefore resembled an aliquot of homogenized cow’s milk, which also showed no separation after centrifugation, but was clearly different to a sample of human donor milk (Fig. 3). The biotechnical analysis was performed in aliquots of the mother’s milk, homogenized cow’s milk, and donor human milk. SDS-Page revealed high casein levels in the mother’s milk aliquots, as usually found in cow’s milk, but not in human breast milk. Liquid chromatography-mass spectrometry confirmed that the milk provided by the mother contained only a minor concentration of actual human breast milk (Fig. 4).

Upon questioning, and in long and careful conversations involving a translator and a psychologist, the mother admitted supplementing her breast milk with cow’s milk. She also admitted to the consumption of high-proof alcohol (vodka), which had been suggested to her by an aunt as a way of building up her strength and enabling her to produce more milk. The mother reported making abnormally heavy use of alcoholic hand sanitizer before providing kangaroo care and milk pumping. However, it has to be noted that ethanol concentrations in breast milk of 2% are not achievable by maternal alcohol consumption, and ethanol is not naturally contained in cow’s milk. Therefore, the high ethanol concentration of 2% can only be explained by a direct admixture of alcoholic beverages. The mother negated this. The mother claimed to have acted on account of making sure her baby gets sufficient nutrition since she could not provide enough milk of her own, and in her home village, it was common for mothers to supplement with cow’s or goat’s milk.

No further episodes of coma and lactic acidosis were observed after converting the infant’s enteral nutrition to hydrolyzed formula and donor human milk. Throughout the further course of the infant’s neonatal care, we maintained close contact with the parents and organized regular conversations, including translators and the psychologist of the NICU. The infant was included in the hospital’s aftercare program with regular visits to the family’s home. Neurodevelopmental follow-up assessments were performed according to the German neonatal guidelines. At the age of 2 years, we transferred the child to our hospital’s social-pediatric center of our hospital for further care and observation of development. Fortunately, the child had only moderate gross and fine motor development delays, but distinct delays in speech development. In the Bayley Scales of Infant Development III (BSID) at the age of 25 months (corrected for gestational age) the child achieved a composite score of 55 for cognitive, 48 for language and 76 for motor performance, corresponding to an age equivalent of 17, 14 and 21 months.

She received logopedic and physical therapy. Physically the infant showed normal growth and weight gain. However, there is marked microcephaly (43 cm at 2 years, z-score − 4.21). This is a known symptom of fetal alcohol syndrome complex. Nonetheless, it might also have developed in the context of extreme premature birth and long-term non-invasive ventilation via face masks and hats, which might influence the head shape.

## Discussion

Neonatal ethanol intoxications are scarce. In one case, a 15-day old infant was admitted to the hospital after accidental preparation of formula with alcohol instead of water [1]. Another small case series reports neonatal ethanol intoxications by regular alcohol intake of the breastfeeding mothers [9]. However, to our knowledge, this is the first report on severe life-threatening ethanol intoxications by ingestion of alcoholized milk in an in-hospital setting.

Intake of alcohol by the nursing child can have an impact on the infant’s behavior and sleep [2]. Another study demonstrated an adverse effect of alcohol in the breastfeeding period on the children’s development after 1 year [10]. Interestingly, the follow-up study in the same cohort, could not confirm this effect after 18 months [11]. It has been suggested, that mothers who consume alcohol after birth, often have already done so during pregnancy [4]. To determine, whether impaired developmental outcome can be assigned to postnatal alcohol intake is therefore difficult and has to be carefully examined in further observations, since ethanol metabolism in newborns is not yet fully understood [8]. Supplementing her own milk with cow’s milk did probably not cause major complications in this specific case. It is important to note, that infants with a low birthweight require a specific and safe nutrition to cover their energy and micronutrient needs [12].

According to the public information website of the center for disease control (CDC) alcohol levels are usually highest in breast milk 30–60 minutes after an alcoholic beverage is consumed, and can be generally detected in breast milk for about 2–3 hours per drink after it is consumed. Although moderate alcohol consumption by a breastfeeding mother (up to 1 standard drink per day) is not known to be harmful to the infant, especially if the mother waits at least 2 hours after a single drink before nursing, not drinking alcohol is the safest option for breastfeeding mothers [13, 14].

## Conclusion

The acute episodes of tachycardia, apnea, and coma in an extremely premature infant were identified as severe ethanol intoxications due to alcoholized breastmilk, possibly aggravated by transcutaneous absorption of alcoholic hand sanitizer. Cultural differences and high language barrier due to the family’s background were the main causes of these life-threatening events. Close observations of the symptoms and visiting habits of the mother lead to identifying a pattern in the described episodes. This resulted in the suspicion of intoxications and further diagnostic workup confirmed our suspicions and, eventually, saved the preterm’s life. Establishing a confidential relationship and communication between parents and the medical staff is very important in the holistic care of infants in the NICU. Therefore, resources must be expanded to ensure communication between health care providers and parents. Family history or specific cultural habits need to be clarified to avoid adverse events. The parents were not aware that formula is plenty in developed countries, and in some hospitals, even human donor milk is available for sick neonates. Thus, her efforts of supplementing her milk were not vital but, in this case, even life-threatening. We assumed that the family was aware of that, as the parent’s consent was given to this precedingly when they were educated after birth, then translated by the father. We understand that this is a very special case but want to point out the relevance of awareness to child safeguarding issues in neonates even in in-hospital environments.

We also emphasize the importance of established structures providing professional translations, especially in the treatment of critically ill patients considering cultural factors.

## Data Availability

All data are clinical and have been obtained from the patient’s chart. Therefore, no datasets were generated or analyzed.

## Abbreviations

BSID: Bayley Scales of Infant Development
NICU: Neonatal intensive care unit
NO: Nitrogen monoxide
ETG: Ethylglucuronide
RBCs: Red blood cells
SDS-Page: Sodium dodecyl sulfate polyacrylamide gel electrophoresis

## References

[1] Zaitsu M, Inada Y, Tashiro K, Hayashi C, Doi H, Hamasaki Y (2013). Acute alcohol intoxication in a 15-day-old neonate. Pediatr Int.

[2] Haastrup MB, Pottegård A, Damkier P (2014). Alcohol and breastfeeding. Basic Clin Pharmacol Toxicol.

[3] Minera G, Robinson E (2014). Accidental acute Alcohol intoxication in infants: review and case report. J Emerg Med.

[4] May PA, Hasken JM, Blankenship J, Marais AS, Joubert B, Cloete M (2016). Breastfeeding and maternal alcohol use: prevalence and effects on child outcomes and fetal alcohol spectrum disorders. Reprod Toxicol.

[5] Mennella J (2001). Alcohol’s effect on lactation. Alcohol Res Heal J Natl Inst Alcohol Abus Alcohol.

[6] Christiansen N (2015). Ethanol exposure through medicines commonly used in paediatrics. Arch Dis Child - Educ Pract.

[7] Hsieh S, Sapkota A, Wood R, Bearer C, Kapoor S. Neonatal ethanol exposure from ethanol-based hand sanitisers in isolettes. Archives of Disease in Childhood: Fetal and Neonatal Edition. 2018;103(1):F1–4. 10.1136/archdischild-2016-311959. 10.1136/archdischild-2016-311959 28588125

[8] Marek E, Kraft WK (2014). Ethanol Pharmacokinetics in Neonates and Infants. Curr Ther Res - Clin Exp.

[9] Hon KL, Wong YCT, Chau IKY, Chau MKT, Cheung KL, Wong W (2016). Alcohol exposure in breastfed neonates associated with Chinese chicken wine. Indian J Pediatr.

[10] Little RE, Anderson KW, Ervin CH, Worthington-Roberts B, Clarren SK (1989). Maternal alcohol use during breast-feeding and infant mental and motor development at one year. N Engl J Med.

[11] Little RE, Northstone K, Golding J, Team AS (2002). Alcohol, breastfeeding, and development at 18 months. Pediatrics.

[12] Dutta S, Singh B, Chessell L, Wilson J, Janes M, McDonald K (2015). Guidelines for feeding very low birthweight infants. Nutrients.

[13] Eidelman AI, Schanler RJ, Johnston M, Landers S, Noble L, Szucs K, Viehmann L. Breastfeeding and the Use of Human Milk. Pediatrics March. 2012;129(3):e827–41. 10.1542/peds.2011-3552. 10.1542/peds.2011-3552 22371471

[14] Sachs HC. The transfer of drugs and therapeutics into human breast milk: an update on selected topics. Pediatrics. 2013;132(3). 10.1542/peds.2013-1985 23979084

